# In Parkinson’s disease on a probabilistic Go/NoGo task deep brain stimulation of the subthalamic nucleus only interferes with withholding of the most prepotent responses

**DOI:** 10.1007/s00221-015-4531-2

**Published:** 2016-01-12

**Authors:** Dejan Georgiev, Georg Dirnberger, Leonora Wilkinson, Patricia Limousin, Marjan Jahanshahi

**Affiliations:** Sobell Department of Motor Neuroscience and Movement Disorders, UCL Institute of Neurology, 33 Queen Square, London, WC1N 3BG UK; Department of Clinical Neuroscience, Danube University, Dr.-Karl-Dorrek-Straße 30, 3500 Krems, Austria; Behavioural Neurology Unit, National Institute of Neurological Disorders and Stroke, National Institutes of Health, 10 Centre Dr., MSC 1440, Bethesda, MD 20892-1440 USA

**Keywords:** Subthalamic nucleus (STN), Deep brain stimulation (DBS), Parkinson’s disease (PD), Go/NoGo task, Prepotency, Load-dependent effects

## Abstract

The evidence on the impact of subthalamic nucleus deep brain stimulation (STN-DBS) on action restraint on Go/NoGO reaction time (RT) tasks in Parkinson’s disease (PD) is inconsistent; with some studies reporting no effect and others finding that STN stimulation interferes with withholding of responses and results in more commission errors relative to STN-DBS off. We used a task in which the probability of Go stimuli varied from 100 % (simple RT task) to 80, 50 and 20 % (probabilistic Go/NoGo RT task), thus altering the prepotency of the response and the difficulty in withholding it on NoGo trials. Twenty PD patients with STN-DBS, ten unoperated PD patients and ten healthy controls participated in the study. All participants were tested twice; the order of on versus off stimulation for STN-DBS PD patients was counterbalanced. Both STN-DBS and unoperated PD patients were tested on medication. The results indicated that STN-DBS selectively decreased discriminability when the response was most prepotent (high—80 %, as compared to low Go probability trials—50 and 20 %). Movement times were faster with STN stimulation than with DBS off across different Go probability levels. There was neither an overall nor a selective effect of STN-DBS on RTs depending on the level of Go probability. Furthermore, compared to healthy controls, both STN-DBS and unoperated PD patients were more prone to making anticipatory errors; which was not influenced by STN stimulation. The results provide evidence for ‘load-dependent’ effects of STN stimulation on action restraint as a function of the prepotency of the Go response.

## Introduction

Deep brain stimulation (DBS) of the subthalamic nucleus (STN), (STN-DBS), is an effective treatment for the motor symptoms and improves quality of life in Parkinson’s disease (PD) (Deuschl et al. 2006; Williams et al. 2011; Weaver et al. 2012). STN-DBS does not affect global cognitive functioning in PD (Krack et al. 2003; Follett et al. 2010; Williams et al. 2011; Rothlind et al. 2014), and only isolated deficits in verbal fluency have been documented following surgery (Parsons et al. 2006). However, there is evidence that executive control of action becomes worse with STN-DBS in PD (Frank et al. 2007; Jahanshahi 2013; Jahanshahi et al. 2015; Williams et al. 2015). This was shown with various tasks of executive function, such as the Stroop (Jahanshahi et al. 2000; Brittain et al. 2012), fast-paced random number generation (Thobois et al. 2007; Anzak et al. 2011), the stop signal task (Ray et al. 2012; Obeso et al. 2014), the Go/NoGo task (Hershey et al. 2004, 2010; Kühn et al. 2004; Ballanger et al. 2009), the Simon interference task (Wylie et al. 2010); and on tasks requiring decision-making under conflict (Frank et al. 2007; Cavanagh et al. 2011; Coulthard et al. 2012; Zaghloul et al. 2012).

The Go/NoGo task is an established paradigm that requires a motor response to one type of stimulus (Go) but not to another (NoGo). It is used to assess action restraint, i.e. the ability to withhold a response to a prepotent stimulus. By altering the ratio of Go and NoGo trials, the task allows testing different levels of preparedness for performance of a given motor response on ‘Go’ trials which in turn influences the ease/difficulty of withholding it on ‘NoGo’ trials. The Go/NoGo task has been used to assess the effect of STN-DBS on inhibitory processes in a number of studies (Hershey et al. 2004, 2010; Kühn et al. 2004; van den Wildenberg et al. 2006; Ballanger et al. 2009), which are summarized in Table 1. As evident from Table 1, the results of these studies are inconsistent. While Hershey et al. (2004, 2010), as well as Ballanger et al. (2009), reported that STN-DBS disrupted the ability to withhold responses on NoGo trials, van den Wildenberg et al. (2006) did not find such an effect.

**Table 1:** Studies investigating the effect of subthalamic nucleus deep brain stimulation (STN-DBS) on Go/NoGo task performance in PD

References	Med. status	*N*	Type of Go/NoGo task	Go trials probability	Commission error rate	Summary of results
Hershey et al. (2004)	Off	24 STN-DBS PD	Letter/number Non-lateralized Response button press	50 and 83 % CRT	NR	STN stimulation significantly reduced discriminability on high (83 %) CRT only
Kühn et al. (2004)	Off	8 STN-DBS PD	Letter/number Lateralized Response button press	80 % CRT	NR	No effect of stimulation on either error rates (commission, omission and laterality error rates) or RT
van den Wildenberg et al. (2006)	On	17 STN-DBS PD 15 Vim-DBS PD	Green/red arrow Non-lateralized Response button press	50 % CRT	0.034 (STN-DBS ON) 0.030 (STN-DBS OFF)	No effect of stimulation on either false alarms or RT
Ballanger et al. (2009)	Off	7 STN-DBS PD	Circle/X Non-lateralized Response button press	100 % SRT and 60 % CRT	0.04 (STN-DBS OFF) 0.10 (STN-DBS ON)	STN stimulation significantly reduced RT and increased the commission error rate
Hershey et al. (2010)	Off	10 STN-DBS PD	Letter/number Non-lateralized Response button press	83 % CRT	0.84 (STN-DBS OFF) 0.82 (dorsal STN-DBS ON) 0.78 (ventral STN-DBS ON)	Ventral STN stimulation reduced hits and increased false alarms, i.e. decreased discriminability, but did not affect RT

*Med. status* medication status (*off* off medication, *on* on medication), *N* number of participants, *NR* not reported, *STN-DBS ON* STN-DBS on stimulation, *RT* reaction time, *STN-DBS OFF* STN-DBS off stimulation, *SRT* simple reaction time, *CRT* choice reaction time

Hershey et al. (2004) tested whether STN-DBS in PD affects action restraint depending on the level of demand on cognitive control. They used a Go/NoGo task with two levels of Go probability (i.e. two levels of prepotency)—83 and 50 %. The results showed that PD patients with STN-DBS were less able to discriminate between Go and NoGo trials on the 83 % Go task (when the response was more prepotent and with high demand on cognitive control) with the stimulation on. This was not observed for the 50 % Go task (low demand on cognitive control). More recently, using a paced random number generation (RNG) task at different rates, with faster rates requiring a higher level of executive control, Williams et al. (2015) showed that STN-DBS differentially impaired inhibition of habitual counting responses during paced RNG in a load-dependent fashion and only at the fastest rate of 1 Hz. Patients could switch to more controlled RNG strategies during conditions of low cognitive load at slower rates only when the STN stimulators were off, but when STN stimulation was on, they engaged in more automatic habitual counting under increased cognitive load at the fastest rate.

In the light of the results of Hershey et al. (2004) and Williams et al. (2015), the first aim of the study was to investigate the effect of STN-DBS on action restraint as a function of the prepotency of the Go response. We compared tasks with three different levels of prepotency of the Go response (probabilistic Go/NoGo reaction time (RT) task—pGNG—with 80, 50 and 20 % Go stimuli). As outlined above, our prediction was that for PD patients with STN-DBS, stimulation would selectively impair action restraint and result in lower discriminability in conditions where the Go response is most prepotent, that is in the 80 % Go task.

In a probabilistic choice RT saccade task, Antoniades et al. (2014) recently reported that, in contrast to healthy controls, six PD patients with STN-DBS on did not show increased reaction time as target probability decreased. The prolongation of RT with the decrease of the target probability is a normal behavioural adjustment, and this was observed with STN-DBS off but not on. These results were explained as an STN-dependent normalization of the neural representation of prior probabilities that is disrupted by STN-DBS. However, somewhat different results were obtained by Obeso et al. (2009) albeit in a single PD patient with a combined unilateral left subthalamotomy and pallidotomy which effectively disrupted left basal ganglia output. On a probabilistic Go/NoGo task, Obeso et al. (2009) found that relative to the ipsilesional left hand, RTs with the right hand contralateral to the left subthalamotomy and pallidotomy were significantly faster on blocks of trials with high (100 and 80 %), but non-significantly slower on blocks with lower probability (50 and 20 %) of Go stimuli. Therefore, the second aim of our study was to explore the effect of STN-DBS on changes in RT across the four levels of Go stimulus probability (100 %—simple RT, and 80, 50 and 20 % pGNG).

## Methods

### Participants

Twenty PD patients with bilateral STN-DBS, ten unoperated PD patients (PD controls) and ten age-matched healthy participants took part. The demographic and clinical data, including total L-dopa equivalence dose—LEDD, LEDD calculated from the L-dopa medication (L-dopa LEDD), LEDD calculated form dopaminergic agonists (DA-LEDD) (Tomlinson et al. 2010), Unified Parkinson’s disease Rating Scale—UPDRS (Fahn and Elton 1987) on and off stimulation and group summary of the stimulation parameters, are presented in Table 2. The MRI-guided surgical approach was used to implant the DBS electrodes in STN-DBS PD patients (Foltynie et al. 2011). The joint ethics committee of the UCL Institute of Neurology and the National Hospital for Neurology and Neurosurgery approved the study. Informed consent was obtained from all participants.

**Table 2:** Main demographic and clinical data for the participants in the study

	STN-DBS PD (*SD*)	PD controls (*SD*)	HC (*SD*)
Age	56.77 (8.93)	57.70 (7.76)	54.00 (7.09)
Sex (f/m)	12/8	4/6	6/4
Handedness (r/l)	18/2	10/0	9/1
Years of education	13.18 (2.42)	13.50 (2.41)	14.20 (2.74)
Disease duration (years)	15.27 (4.34)	13.30 (5.54)	–
Time since operation	3.30 (1.25)	–	–
LEDD (total)	575.86 (195.14)	688.31 (249.82)	–
L-dopa LEDD	285.34 (161.12)	354.21 (220.54)	–
DA-LEDD	275.12 (145.15)	310.12 (113.17)	–
UPDRS on med/off stim.	24.09 (10.42)	21.75 (8.45)	–
UPDRS on med/on stim.	12.55 (8.81)	–	–
Median frequency (r/l)	130/130 Hz	–	–
Median pulse width (r/l)	60/60 μs	–	–
Mean amplitude (r/l)	2.99 (0.71)/3.27 (0.76) V	–	–

Mean values and standard deviations (*SD*, given in brackets) or median values (where appropriate) are presented

*f/m* number of females/males, *r/l* right/left, *LEDD* Levodopa equivalent daily dose, *DA* dopaminergic agonists, *STN* subthalamic nucleus, *PD* Parkinson’s disease, *HC* healthy control participants, *UPDRS* Unified Parkinson’s Disease Rating Scale, Median frequency of STN-DBS in Hertz (Hz), Median DBS pulse width in microseconds (μs) and Mean DBS amplitude in volts (V)

### Study design

A mixed-factorial repeated-measures design was used. All participants performed the task twice (30 min interval). The order of assessment on versus off stimulation was counterbalanced in the PD patients with STN-DBS. All PD patients performed the task on medication at least 30 min, but not longer than 90 min after taking their usual drugs.

### Go/NoGo task

**Fig. 1:**
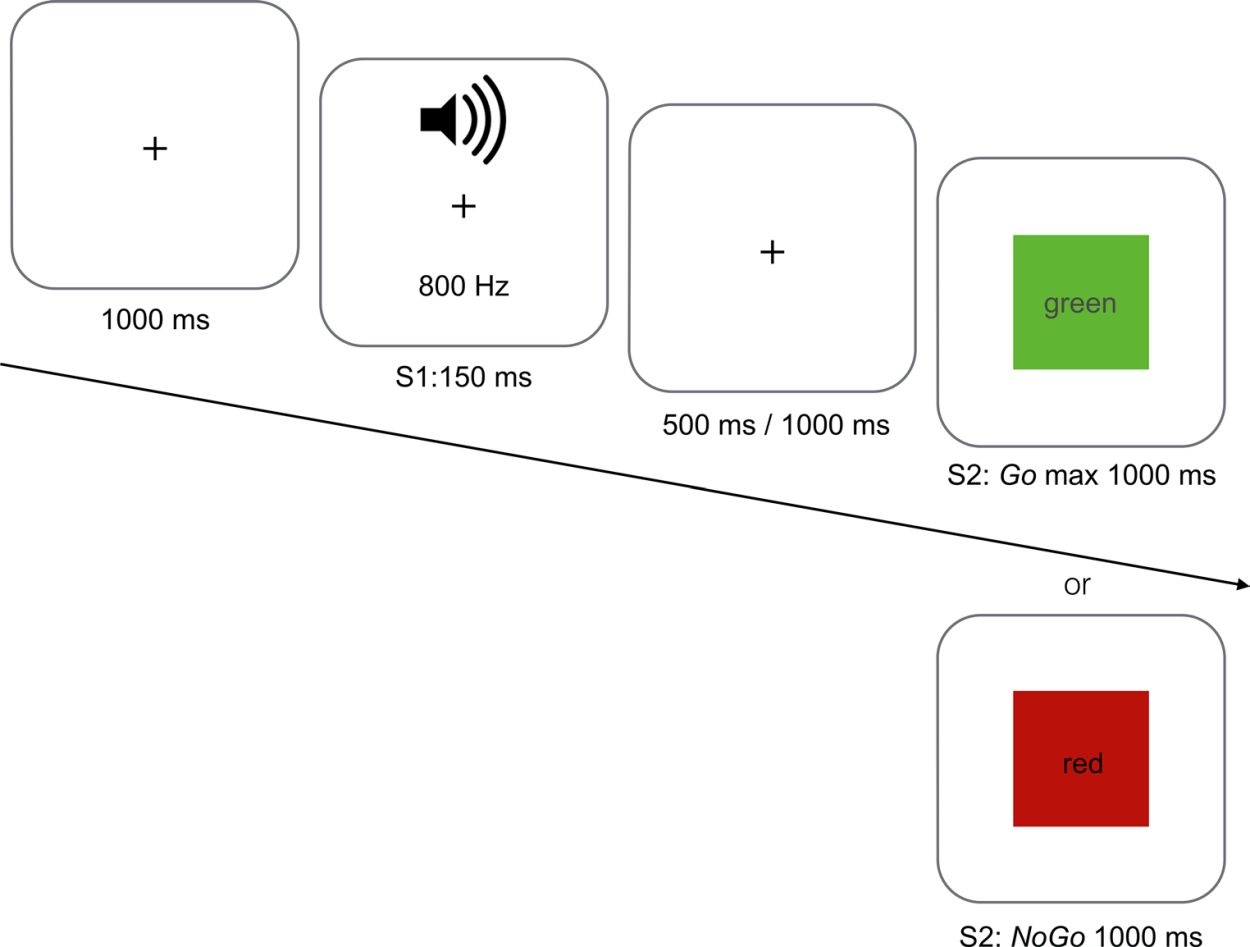
Schematic representation of the Go/NoGo task used in the study. As soon as the home key was pressed, a fixation cross was presented on the monitor for 1000 ms; this was followed by the auditory warning stimulus S1. After a variable interval of 500 or 1000 ms, the visual S2 stimulus was presented (*Go*—*green square*, or NoGo—*red square*). There were four different blocks with four levels of Go stimulus probability levels—100 % (SRT), 80 % (pGNG80), 50 % (pGNG50) and 20 % (pGNG20). For details, please see “Methods” (color figure online)

Participants were tested on a simple reaction time task (SRT, thus Go probability level of 100 %) and on a probabilistic Go/NoGo reaction time task (pGNG) with three different levels of Go stimulus probabilities (80, 50 and 20 % Go trials; pGNG80, pGNG50 and pGNG20, respectively). These conditions were tested in four experimental blocks of 100 trials each (Fig. 1). An S1 auditory warning stimulus (800 Hz, 150 ms) was followed by a variable 500- or 1000-ms interval. This was followed by the visual S2 stimulus—in the SRT task always a Go stimulus (green square), in pGNG tasks by the same Go stimulus or a NoGo stimulus (red square). In all blocks, participants were instructed to press and hold down a home key (black button) until the Go signal was presented on the screen. This was the signal to release the home key, move their hand and press the response key (green button). In NoGo trials, participants were instructed to withhold any response on presentation of the red square and continue to hold down and press the home key.

Participants were instructed to always respond with their dominant hand. On the Go trials, the S2 disappeared immediately after pressing the response key. If they failed to move the finger from the home key (“no response”), the S2 disappeared after 1000 ms. On the NoGo trials, the S2 always disappeared after 1000 ms.

### Data analysis

The reaction time was defined as the interval between the onset of S2 and the participant lifting the index finger from the home key. The movement time (MT) was defined as the interval between the participant lifting the index finger from the home key and pressing the response key. While RT is a measure of the time needed to make a decision, MT is a measure of the time needed for movement execution (Jensen 1987). The distinction between RT and MT is important, as the compound measure (response time) that is usually used in reaction time tasks, takes into account both the RT and MT, and might not therefore reflect the speed of cognitive processes during task execution.

*Anticipatory errors* were defined as Go trials with correct responses and RTs shorter than 100 ms. *Commission errors* were trials when the participants lifted their finger from the home key on NoGo trials (i.e. the trials on which they failed to withhold a response on NoGo trials). *Omission errors* were Go trials on which the participant did not lift the finger from the home key (i.e. Go trials on which they failed to respond). Because the rate of omission errors was very low in all three groups of participants in all conditions, these were excluded from further analysis.

The following relative rates were computed and compared in the subsequent analysis: commission error rate (CER), hit rate (HR) and anticipation error rate (AnER). CER was defined as the proportion of commission errors out of the total number of NoGo trials in a task. The HR was defined as the proportion of Go trials with correct responses out of the total number of Go trials. AnER was the proportion of anticipation errors out of the total number of Go trials.

The discriminability index (Pr), derived from signal detection theory (Macmillan and Creelman 2005), was calculated as the difference between the hit rate and the commission error rate (Pr = HR-CER). According to the signal detection theory, Pr is a measure of how well a person can discriminate between the two choices (Hershey et al. 2010) and it is therefore suitable to be calculated in a Go/NoGo task in which participants have to discriminate between the possibility to respond on presentation of one stimulus (Go) and withhold the response on presentation of another stimulus (NoGo).

### Statistical analysis

SPSS was used for statistical analysis of the data. The Shapiro–Wilk test was used to test for normality. Parametric data were analysed with repeated-measures ANOVA, mixed-design ANOVA and *t* test. If the assumption of sphericity was violated, the Greenhouse–Geisser correction was used. Chi-square tests were used to analyse nonparametric data. A probability value of *p* = 0.05 was set to test for significance. Bonferroni corrections were used to control for multiple comparisons.

## Results

There was no difference in age (*F*(2) = 2.78, *p* = 0.378), handedness (*χ*^2^(2) = 2.22, *p* = 0.329), gender (*χ*^2^(2) = 1.49, *p* = 0.567) or years of education (*F*(2) = 0.52, *p* = 0.597) between the STN-DBS PD patients, PD controls and healthy controls. There was also no difference in LEDD (*t*(28) = −0.51, *p* = 0.617) or mean disease duration (*t*(28) = 1.84, *p* = 0.182) between the STN-DBS PD patients and unoperated PD controls. As expected, the motor part of the UPDRS was higher in PD patients on medication compared to STN-DBS PD on medication and on stimulation, *t*(28) = 2.29, *p* = 0.035. Similarly, the motor symptoms of the STN-DBS PD patients on medication and on stimulation were significantly improved compared to on medication and off stimulation (*t*(19) = 4.54, *p* = 0.001) (Table 2).

Before the mixed repeated-measures factorial model with between-subject factor ‘group’ (STN-DBS PD, PD controls and healthy controls), and within-subject factors ‘stimulation’/time of assessment (STN-DBS on vs. STN-DBS off or first vs. second assessment) and ‘probability level’ of Go trials (SRT, pGNG80, pGNG50 and pGNG20) was applied to the various measures (RT, MT, HR, CER, AnER and Pr), the task performance for the first versus second assessment was compared for the unoperated PD patients and healthy controls. There was no difference in task performance on any of the behavioural measures (i.e. RT, MT, HR, CER, AnER, and Pr) between the first and the second assessment for either the unoperated PD (all *p*s > 0.05) or the healthy controls (all *p* > 0.05). This means that there were no carry-over effects between the first and second assessment, and therefore, the data from the first and second assessment for the unoperated PD patients and healthy controls are equivalent and can be used interchangeably in comparison with the data from patients with STN-DBS.

### STN stimulation decreased discriminability in the pGNG80 task only

**Fig. 2:**
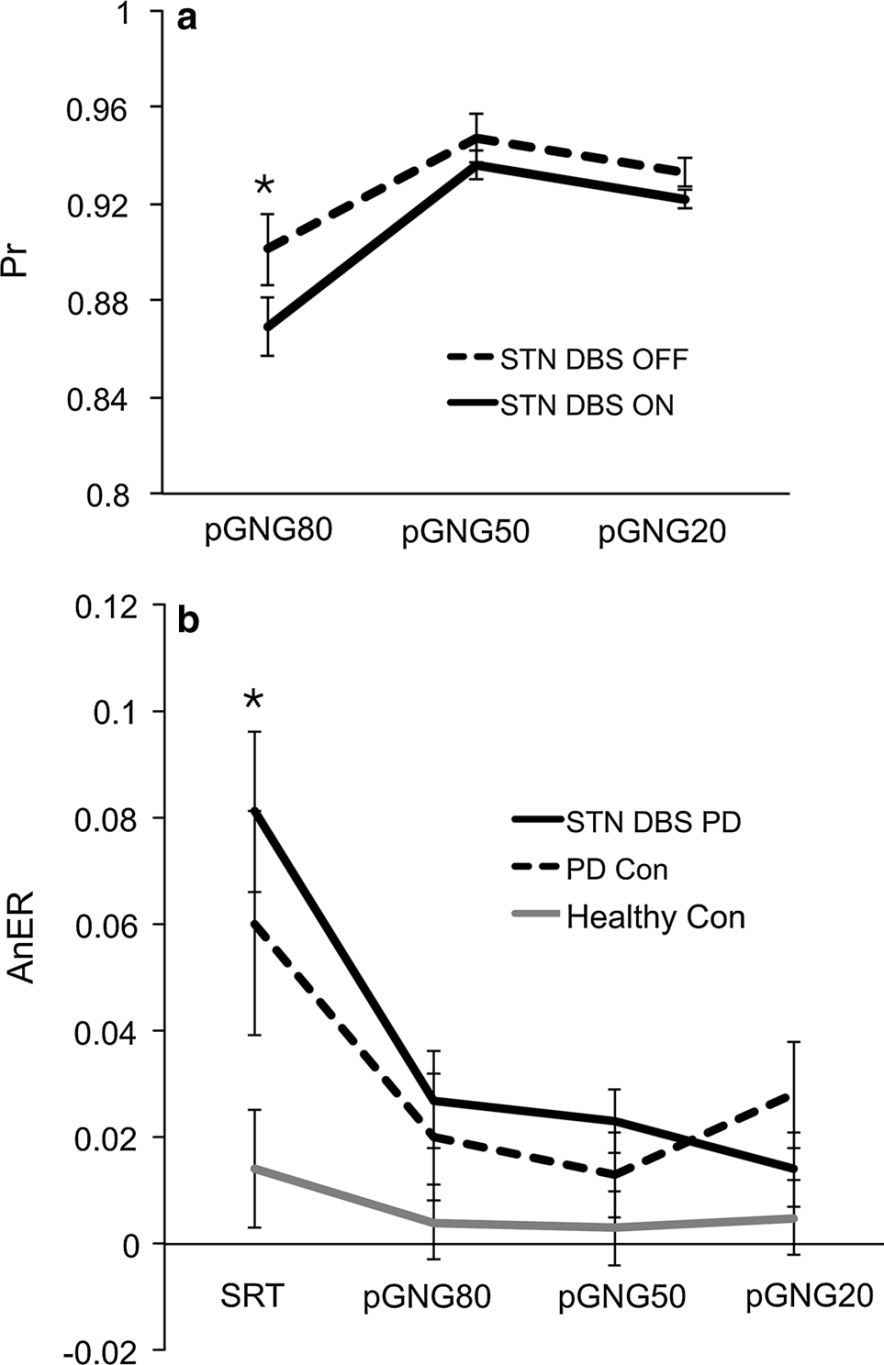
Significant interaction effects for the discriminability index (Pr) (**a**) and anticipatory error rate (AnER) (**b**) for PD patients with STN-DBS (PD STN-DBS), unoperated PD (PD Con) and healthy controls (Con) for four different levels of Go stimulus probability levels—100 % (SRT), 80 % (pGNG80), 50 % (pGNG50) and 20 % (pGNG20). *DBS ON* on stimulation, *DBS OFF* off stimulation. The asterisk (*) indicates significant difference (*p* < 0.05)

As predicted, the discriminability index was the lowest in the task with the Go/NoGo task with highest Go probability level (80 %) (*F*(2,74) = 21.11, *p* < 0.0001) (Table 3). Even though the main effects of stimulation (*F*(1,37) = 0.98, *p* = 0.342) and group (*F*(1,37) = 1.30, *p* = 0.284) were not significant, the significant stimulation × probability interaction (*F*(2,74) = 3.98, *p* = 0.023) (Fig. 2a) indicated a differential effect of stimulation depending on the Go probability. Indeed, while the discriminability index on stimulation was lower compared to off stimulation (*F*(1,37) = 3.90, *p* = 0.042) for the 80 % Go probability task (pGNG80), there was no significant difference in the discriminability index on versus off stimulation for the 50 % (pGNG50) or 20 % (pGNG20) Go probability tasks (*F*(1,39) = 1.24, *p* = 0.272). On the pGNG80 task, 14 of the 20 (70 %) patients showed decreased discriminability index on versus off STN stimulation. On the pGNG50, only four patients (20 %) showed decreased discriminability index on versus off STN-DBS. For the pGNF20 task, seven patients (35 %) showed decreased discriminability on compared to off STN stimulation. There were no significant correlations of the discriminability index on or off stimulation (derived for all three tasks pGNG80, pGNG50, pGNG20) with RT, UPDRS, LEDD, age, disease duration or amplitude of stimulation (all *p*s > 0.05).

**Table 3:** Mean values and standard error of the mean (SEM) in brackets for the hit rate, commission error rate, anticipation error rate and discriminability (Pr) index for PD patients with STN-DBS ON and OFF, PD control patients (PD Con) and healthy controls (HC) 1st and 2nd assessment in all tasks: simple reaction time (SRT), and probabilistic Go/NoGo task with three levels of Go signal probability—pGNG80, pGNG50 and pGNG20

	PD STN-DBS ON	PD STN-DBS OFF	PD Con 1st	PD Con 2nd	HC 1st	HC 2nd
*Hit rate*						
SRT	0.976 (0.010)	0.984 (0.004)	0.989 (0.006)	0.990 (0.003)	0.980 (0.001)	0.990 (0.003)
pGNG80	0.973 (0.004)	0.979 (0.005)	0.987 (0.002)	0.975 (0.005)	0.988 (0.002)	0.986 (0.005)
pGNG50	0.969 (0.006)	0.978 (0.001)	0.960 (0.002)	0.977 (0.006)	0.980 (0.003)	0.983 (0.006)
pGNG20	0.935 (0.007)	0.945 (0.003)	0.950 (0.002)	0.950 (0.002)	0.952 (0.002)	0.954 (0.002)
*Commission error rate*						
pGNG80	0.110 (0.025)	0.078 (0.029)	0.075 (0.024)	0.118 (0.037)	0.073 (0.023)	0.105 (0.003)
pGNG50	0.037 (0.007)	0.031 (0.008)	0.039 (0.009)	0.020 (0.006)	0.018 (0.006)	0.021 (0.006)
pGNG20	0.013 (0.003)	0.012 (0.002)	0.015 (0.005)	0.020 (0.007)	0.008 (0.003)	0.007 (0.002)
*Anticipation error rate*						
SRT	0.090 (0.017)	0.073 (0.020)	0.067 (0.002)	0.052 (0.016)	0.020 (0.006)	0.009 (0.003)
pGNG80	0.042 (0.003)	0.025 (0.009)	0.010 (0.003)	0.030 (0.009)	0.005 (0.001)	0.003 (0.001)
pGNG50	0.034 (0.004)	0.015 (0.007)	0.006 (0.001)	0.020 (0.006)	0.003 (0.001)	0.006 (0.002)
pGNG20	0.018 (0.003)	0.013 (0.003)	0.020 (0.006)	0.035 (0.011)	0.005 (0.002)	0.005 (0.002)
*Discriminability (Pr)*						
pGNG80	0.863 (0.029)	0.901 (0.029)	0.912 (0.026)	0.857 (0.034)	0.915 (0.011)	0.881 (0.037)
pGNG50	0.932 (0.090)	0.947 (0.009)	0.921 (0.020)	0.957 (0.010)	0.962 (0.002)	0.962 (0.012)
pGNG20	0.922 (0.004)	0.933 (0.004)	0.935 (0.009)	0.930 (0.008)	0.944 (0.009)	0.947 (0.002)

The main effect of stimulation/time of assessment was not significant for either the HR (*F*(1,37) = 0.82, *p* = 0.370) or the CER (*F*(1,37) = 1.50, *p* = 0.228). There was also no significant effect of group for either the HR (*F*(1,37) = 2.43, *p* = 0.102) or the CER (*F*(1,37) = 0.96, *p* = 0.391). As expected, while the HR was the lowest in pGNG20 (*F*(3,111) = 55.23, *p* < 0.0001), the CER was highest in the task with the highest probability of Go responses (pGNG80) (*F*(2,74) = 39.95, *p* < 0.0001) (Table 3). There were no significant interaction effects for either HR or CER.

### Both STN-DBS and unoperated PD patients made more anticipation errors than healthy controls

The main effect of stimulation/time of assessment was not significant (*F*(1,37) = 1.83, *p* = 0.185). As expected, the highest AnER was associated with the highest probability of Go signal presentation in the SRT (*F*(3,111) = 10.75, *p* < 0.0001). The significant main effect of group (*F*(1,37) = 3.59, *p* = 0.040) indicated higher AnER for STN-DBS PD patients and PD controls compared to healthy participants (Table 3). Furthermore, the significant group × probability interaction (*F*(6,111) = 2.55, *p* = 0.024) (Fig. 2b) indicated that the difference in AnER between groups was due to the difference in AnER for the SRT task (*F*(6,37) = 3.36, *p* = 0.047) mainly due to higher anticipatory errors for the STN-DBS PD patients than the healthy controls (*F*(1,28) = 6.61, *p* = 0.016) but also due to higher anticipations for the unoperated PD compared to healthy controls (*F*(1,18) = 4.46, *p* = 0.049). The difference between STN-DBS PD patients and PD controls was not significant (*F*(1,28) = 0.53, *p* = 0.470).

### STN stimulation decreased movement time but did not affect reaction time

**Fig. 3:**
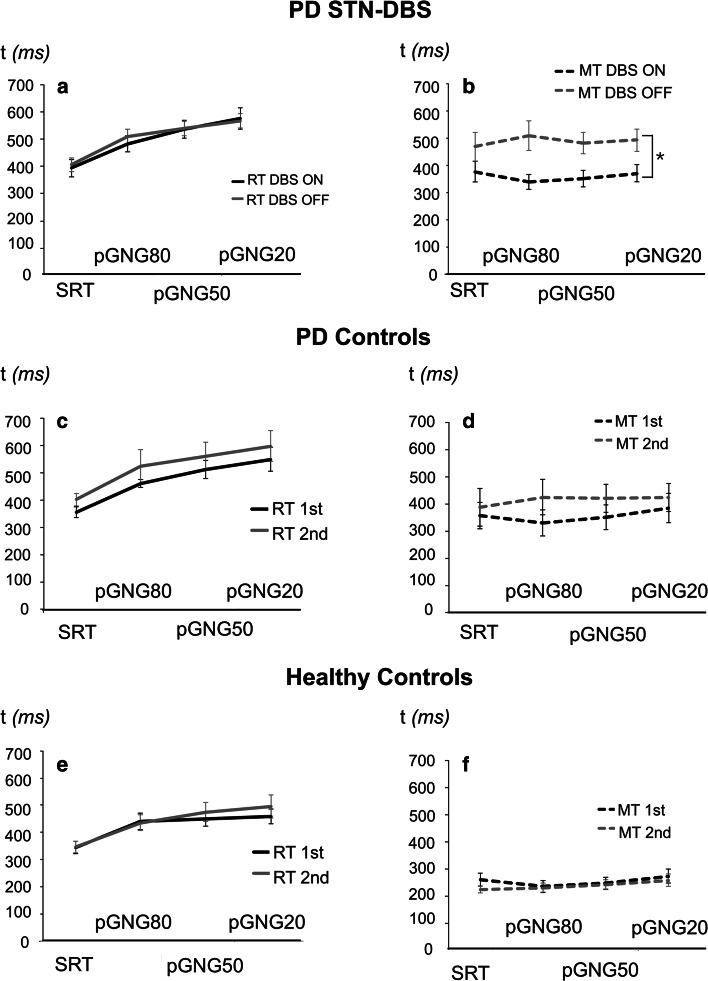
Reaction times (RT) and movement times (MT) for the correct *Go* responses for patients with STN-DBS (PD STN-DBS) (**a**, **b**), PD controls (**c**, **d**) and healthy controls (**e**, **f**) for four different levels of Go stimulus probability levels—100 % (SRT), 80 % (pGNG80), 50 % (pGNG50) and 20 % (pGNG20). *DBS ON* on stimulation, *DBS OFF* off stimulation, *1st* first assessment, *2nd* second assessment. The asterisk (*) indicates significant difference (*p* < 0.05)

MT was shorter on stimulation than with stimulation off (*F*(1,37) = 4.01, *p* = 0.047) (Fig. 3b). As expected, MT was the shortest for healthy controls, followed by PD with STN-DBS and then unoperated PD patients (*F*(1,37) = 7.23, *p* = 0.002) (Fig. 3b, d, f). However, for MT, the main effect of task (*F*(3,111) = 1.78, *p* = 0.156) and the interaction effects were not significant.

For RTs, by contrast to MT, the main effect of stimulation/time of assessment (*F*(1,37) = 2.56, *p* = 0.118) or group (*F*(1,37) = 1.64, *p* = 0.208) was not significant. As expected, across all groups, RT was shortest in the SRT task and longest in pGNG20 (*F*(3,111) = 61.10, *p* < 0.0001, Fig. 3a, c, e). None of the interaction effects were significant (*p* > 0.05).

## Discussion

In summary, STN stimulation had a significant effect on discriminability as a function of the level of prepotency of the response, such that the discriminability on stimulation was selectively lower than off stimulation on the trials with the highest Go probability of 80 % but not for pGNG50 or pGNG20. There was no effect of STN stimulation on RTs. By contrast, MTs were faster on stimulation compared to off stimulation across all tasks (SRT, pGNG80, pGNG50 and pGNG20). In addition, there was a significant effect of group, but not stimulation, on the anticipatory error rate, such that both STN-DBS and unoperated PD patients were more prone to make anticipations as compared to healthy controls.

### STN-DBS disrupts action restraint for the most prepotent responses

Performance on the probabilistic GoNoGo RT tasks depends on a balance between the preparedness to respond on Go trials and the success in withholding responses on NoGo trials (Obeso et al. 2009). The inhibition of a prepared response is most difficult and demanding when the response is more prepotent, that is, in blocks with a higher percentage of Go trials. We therefore predicted that STN-DBS would interfere with action restraint where the Go response was most prepotent, that is on the pGNG80 blocks.

Indeed, the significant stimulation × probability interaction indicated an effect of STN stimulation on discriminability depending on Go probability: PD patients with STN-DBS on stimulation showed the lowest discriminability index in the condition with the highest proportion of Go trials (80 %) compared to lower Go probability (50 and 20 %) on the pGNG. As evident from Table 3, this pattern of results seems to be driven primarily by the stimulation-induced increase in commission errors on NoGo trials since the hit rates do not differ between the two stimulation conditions. Therefore, this suggests that the lowered discriminability is due to increased commission errors, i.e. an inability to withhold responses on the most prepotent 80 % Go/NoGo condition. This supports our first hypothesis and is in line with the findings of Hershey et al. (2004) who found a decrease of discriminability on Go/NoGo tasks with high (83 % Go) but not low (50 %) Go probability. The finding that the discriminability with STN stimulation was lowest on the block with the highest Go probability means that STN-DBS interfered with action restraint on trials when the Go response was most prepotent. Therefore, the results of our study and those of Hershey et al. (2004) and Williams et al. (2015) indicate that the effect of STN-DBS on performance of attention-demanding tasks requiring the ability to withhold prepotent responses is load-dependent. For example, Williams et al. (2015) found that STN-DBS differentially impaired inhibition of habitual counting responses (count score 1) during paced RNG in a load-dependent fashion only at the fastest rate of 1 Hz, but not at the slower rates of 0.33 and 0.5 Hz.

Kühn et al. (2004) recorded local field potentials from the STN in the immediate post-operative phase while PD patients performed a Go/NoGo task. They found that whereas on Go trials, prior to movement, there was a beta desynchronization followed by a late post-movement beta synchronization, on NoGo trials the beta desynchronization was prematurely terminated and immediately reversed into beta synchronization. This was interpreted as indicating that beta activity recorded from the STN may be a marker of initiation (or withholding) of movement programming. The imaging study of Ballanger et al. (2009) highlighted the brain networks that were modulated by STN-DBS in PD during performance of a Go/NoGo task. Relative to DBS off, STN stimulation was associated with a significant reduction of activation in the pre-SMA and dorsal and ventral premotor cortex, dorsal anterior cingulate cortex, primary motor cortex and the inferior frontal cortex, together with an increased activation in the subgenual anterior cingulate cortex. These results were interpreted as STN-DBS modulating networks associated with proactive and reactive inhibition.

In the present study, STN stimulation decreased the HR and increased the CER, but these effects were not significant. There was, however, a significant main effect of probability on both of these measures. HR was lowest on the trials with the lowest level of response prepotency—pGNG20 task, whereas CER was highest during the highest level of response prepotency—pGNG80 task.

### STN-DBS did not disrupt slowing to less probable stimuli

We did not replicate the finding of Antoniades et al. (2014) that STN-DBS disrupted the slowing of RTs to less probable stimuli. Antoniades et al. (2014) used a probabilistic choice RT saccade task (10, 25, 50, 75 and 90 %) to test the effect of STN-DBS on normalization of prior probabilities. They found that when the stimulator was switched off, RTs were shorter for more probable and longer for less probable targets, which is the usual pattern of RT changes as a function of target probability observed in healthy controls as well. In their study, when the STN stimulators were switched on, RTs were still shorter for more probable trials, but they did not increase, as the target became less likely. This was interpreted as indicating that stimulation disrupted the effect of STN on normalization of the neuronal representations of prior probabilities. The fact that Antoniades et al. (2014) tested the effect of STN-DBS on a choice RT saccade task rather than a manual Go/NoGo task could partly account for the discrepancy in findings with our study. The basal–ganglia–thalamo–cortical loops (DeLong 1990; DeLong and Wichmann 2010) are largely segregated, and it is possible that it is easier to observe the effect of normalization of RTs related to prepotency in a saccadic task, involving the ‘oculomotor’ circuit, than in a Go/NoGo task with manual responses mediated by the ‘motor’ circuit. In our study, we recorded both RT and MTs in a probabilistic Go/NoGo RT task as they reflect different processes. While RTs increase proportionally to the amount of information contained in presented stimuli, MT is relatively unaffected by it (Jensen 1987). Therefore, RT corresponds to the time required to make a decision, whereas MT reflects the time needed to execute the response. Indeed, the distinction between RT and MT is one of the advantages of our study. As expected, for all groups the RT was shortest on the trials with the highest level of preparedness to respond, that is during the SRT trials (Go probability of 100 %) and RTs were longest for the lowest probability of Go stimuli (pGNG20). In contrast, the manipulation of Go probability did not have any effect on MT, since MT is simply the time taken for execution of the response. As expected, in our study, MT was the shortest for healthy participants, and importantly, it was shorter for STN-DBS patients with stimulation on than off. The shorter MT on versus off stimulation, which reflects the improved motor abilities of the STN-DBS patients as a result of the stimulation, was also supported by the lower motor UPDRS score for STN-DBS PD patients on than off stimulation. It is possible that in some of the studies that do not distinguish between RT and MT, the effect of STN-DBS on RT (Hershey et al. 2004; Ballanger et al. 2009) is at least partially reflecting movement execution time (MT). Our results of the effect of STN-DBS on RTs as a function of the probability of the Go stimuli is roughly consistent with the performance of a PD patient with a combined subthalamotomy and pallidotomy on the same task as reported by Obeso et al. (2009).

### PD patients made more anticipatory errors than healthy controls

Compared to healthy controls, PD patients, both operated and unoperated patients, were more prone to making anticipatory errors on the trials with the highest level of preparedness, that is on the SRT task (100 % Go probability). Even though the anticipatory error rate was higher for patients with STN-DBS compared to the unoperated PD patients for all blocks except for the lowest Go probability level (pGNG20), this difference was not significant. The increased anticipatory errors for the unoperated patients may reflect executive dysfunction which can be present from the early stages of PD (Dirnberger and Jahanshahi 2013). The increased rate of anticipatory errors in PD may partly relate to the fact that both control PD patients and patients with STN-DBS were tested on medication. Dopaminergic medications are known to adversely affect performance on some tasks in PD (Gotham et al. 1986; Cools 2006). For example, in a recent study Huang et al. (2015) found an increase of the error rate in PD patients on dopaminergic medication compared to the off medication state during a perceptual decision-making task. Moreover, as assessed by the drift diffusion model (Ratcliff and McKoon 2008), which allows for more detailed description of processing in a RT task beyond the accuracy and RT measures by calculating different parameters, such as the boundary separation (the amount of information to reach a decision) and drift rate (speed of information accumulation), the increased error rate in PD patients on medication was a result of a change of the quality of sensory information processing induced by dopaminergic medication that was reflected in a lower drift rate when patients were test on medication relative to the ‘off’ state. The fact that we did not test patients off medication is potentially one of the limitations of the present study. This was primarily because the main objective of this study was to explore the effect of STN-DBS, not the effect of medication, on the probabilistic Go/NoGo task. In addition, we did not test the patients off medication and off stimulation. However, testing PD patients off medication and off stimulation is much more challenging for the patients. However, the fact that we found an effect of STN stimulation on discriminability argues against interference of the on medication state of the patients with STN-DBS during Go/NoGo task execution. Another possible limitation of the study would be that we did not take into account the position of the electrodes. Nevertheless, this issue has already been addressed before in a study by Hershey et al. (2010), who found that even though stimulation of both the ventral and dorsal STN improved motor symptoms in PD, the performance of Go/NoGo task was disrupted by stimulation of the ventral STN only, resulting in lower hits and higher commission errors. On the positive side, relative to most previous studies, which have small samples, in this study we assessed a larger number of patients and compared the performance of STN-DBS PD patients and age-matched unoperated PD patients and healthy participants. We also measured both RTs and MTs and showed that while MTs were shortened with STN-DBS, there was no effect of the STN stimulation on RT.

## Conclusions

STN-DBS selectively decreased discriminability on tasks with high (80 %), but not low probability of Go stimuli (50 and 20 %). Furthermore, while there was an effect of stimulation on MT across different probabilities of Go stimuli, there was no effect of STN stimulation on RT. Compared to healthy controls, both STN-DBS and unoperated PD patients made more anticipatory errors; this was not affected by stimulation. We provide evidence for ‘load-dependent’ effects of STN stimulation as a function of the prepotency of the Go response.
